# Addison’s Disease Presenting with Hypercalcaemia, Acute Kidney Injury, and Anxiety

**DOI:** 10.7759/cureus.101663

**Published:** 2026-01-16

**Authors:** Sonam Tshering, Waqar Qayyum, Ashutosh Kapoor

**Affiliations:** 1 Endocrinology and Diabetes, Barts Health NHS Trust, London, GBR; 2 Medicine, Barts Health NHS Trust, London, GBR; 3 Diabetes and Endocrinology, Health Education England (HEE) North West London, London, GBR

**Keywords:** acute kidney injury, addison's disease, anxiety, parathyroid hormone (pth), pth-independent hypercalcemia, steroid use

## Abstract

This report describes the case of a 41-year-old woman who presented with Addison’s disease, manifesting as hypercalcaemia, acute kidney injury, and anxiety. She experienced progressive weight loss, fatigue, vomiting, dizziness, and anxiety over three months. Initial laboratory investigations revealed parathyroid hormone (PTH)-independent hypercalcaemia and renal impairment. Imaging and biochemical screening excluded malignancy and other common causes of hypercalcaemia. Endocrine evaluation confirmed primary adrenal insufficiency (PAI), evidenced by markedly reduced morning cortisol, elevated adrenocorticotropic hormone (ACTH), and positive adrenal cortex antibodies. Treatment with intravenous fluids and hydrocortisone led to rapid normalisation of calcium and renal function, accompanied by significant improvement in psychiatric symptoms.

## Introduction

Hypercalcaemia is a common electrolyte disturbance, with an overall prevalence of approximately one per 1,000 individuals in the general population and 0.6% in the emergency department [1]. The parathyroid hormone (PTH) test is central to diagnosis, distinguishing between PTH-dependent (normal or high PTH) and PTH-independent (low PTH) forms [2]. Most cases (80% to 90%) are due to primary hyperparathyroidism or malignant disease [1].

Adrenal insufficiency seldom presents with PTH-independent hypercalcaemia [3]. It is estimated that approximately 5.5% to 6% of patients are found to have elevated calcium levels when Addison’s disease is first diagnosed [4]. Adrenal insufficiency is classified as primary (Addison’s disease), secondary (pituitary), or tertiary (hypothalamic), with tertiary often grouped with secondary [5]. Primary adrenal insufficiency (PAI) is characterised by impaired synthesis of glucocorticoids and mineralocorticoids by the adrenal cortex, resulting in a serious and potentially life-threatening disruption of metabolic, electrolyte, and fluid balance [6]. Autoimmune destruction is the main cause of PAI, known as Addison’s disease, which is rare in Europe (about 100 cases per million) [5]. Apart from salt craving, symptoms of PAI are generally non-specific, such as weakness, fatigue, pain, weight loss, abdominal discomfort, depression, and anxiety, which often lead to delayed diagnosis and presentation as a potentially life-threatening adrenal crisis [6]. Despite standard hormone replacement, patients with Addison’s disease often experience reduced quality of life [6]. Furthermore, the longer the delay in diagnosis, the poorer the long-term outcomes, highlighting the need for early recognition [6]. A cross-sectional study of 216 patients with adrenal insufficiency found that fewer than 30% of women and 50% of men were diagnosed within six months of symptom onset, with one in five patients waiting over five years [7]. Most patients consulted at least three physicians, and nearly 70% were initially misdiagnosed, commonly with psychiatric or gastrointestinal conditions [7]. This study also found that patients with adrenal insufficiency had reduced self-perceived health status compared to controls, and those diagnosed within three months had significantly better outcomes [7].

Diagnosis of PAI is confirmed by low morning cortisol (<140 nmol/L) and elevated adrenocorticotropic hormone (ACTH; more than twice the upper limit of normal) [5]; if inconclusive, a cosyntropin stimulation test is recommended [5]. Treatment involves hormone replacement with glucocorticoids and mineralocorticoids.

The occurrence of hypercalcaemia, acute kidney injury, and anxiety together in adrenal insufficiency is uncommon. This case details a patient with Addison’s disease presenting with all three symptoms, emphasising the need to consider adrenal insufficiency in cases of unexplained hypercalcaemia and renal dysfunction to prevent delays in diagnosis.

## Case presentation

A 41-year-old woman presented to the emergency department after three months of deteriorating physical and mental health. Her symptoms included reduced appetite, persistent fatigue, dizziness, intermittent vomiting, and an unintentional weight loss of 19 kilograms. She described her abdominal discomfort as “grumpy.” An anxiety disorder had been diagnosed several months earlier, initially treated with sertraline, which was later switched to citalopram due to diarrhoea.

Her medical history was notable only for hypothyroidism, secondary to radioiodine treatment for Graves’ disease 20 years prior. She was taking levothyroxine and citalopram. There was no family history of autoimmune disease.

On admission, the patient was noted to be hypotensive, with a blood pressure of 88/58 mmHg, and tachycardic, with a heart rate of 100 beats per minute. Her respiratory rate was 17 breaths per minute, and her peripheral oxygen saturation was 100% on room air. Abdominal examination revealed a non-tender abdomen. Her weight was 46 kilograms (BMI of 19 kg/m_2_).

Initial laboratory tests (Table 1) identified normocytic anaemia, lymphocytosis, acute kidney injury, elevated calcium levels and suppressed PTH.

**Table 1:** Laboratory Parameters at Presentation, Baseline, and Six-Month Follow-up Abbreviations: Hb, haemoglobin; WCC, white cell count; MCV, mean cell volume; eGFR, estimated glomerular filtration rate; PTH, parathyroid hormone

Parameter	At Presentation	Baseline (A year ago)	Six-Month Follow-up	Reference Range
Hb (g/L)	116	130	138	120–150
WCC (×10⁹/L)	9.2	9.5	9.7	4–10
MCV (fL)	84.2	—	95.6	83–101
Lymphocyte count (×10⁹/L)	3.2	2.8	4.2	1–3
Serum sodium (mmol/L)	133	140	142	133–146
Serum potassium (mmol/L)	4.8	4.3	4.4	3.5–5.3
Serum urea (mmol/L)	11.7	—	5.5	2.5–7.8
Serum creatinine (µmol/L)	131	75	73	45–84
eGFR (ml/min/1.73m²)	39	75	76	—
Adjusted serum calcium (mmol/L)	2.87	2.42	2.6	2.2–2.6
Phosphate (mmol/L)	1.26	—	—	0.8–1.5
PTH (pmol/L)	0.9	—	—	1.8–7.9
Serum 25-OH, vitamin D (nmol/L)	80	—	—	>50 (sufficient)

In view of PTH-independent hypercalcaemia and significant weight loss, malignancy was considered, but a contrast-enhanced computed tomography (CT) scan of the chest, abdomen, and pelvis revealed no evidence of malignancy, apart from a simple left ovarian cyst. Serum electrophoresis, 25-OH vitamin D levels and angiotensin-converting enzyme (ACE) levels were within normal limits.

By the third day of admission, the endocrinology team evaluated the patient. The combination of ongoing weight loss, frequent vomiting, unexplained hypotension, and negative malignancy screening, along with laboratory findings of low-normal sodium, normocytic anaemia, lymphocytosis, and hypercalcaemia, prompted suspicion of adrenal insufficiency. Hormonal studies (Table 2) revealed a significantly low morning cortisol (7 nmol/L) and markedly elevated ACTH. Positive adrenal cortex antibodies confirmed Addison’s disease. In retrospect, the patient recalled mild skin darkening, though no obvious pigmentation of the skin or mucous membranes was detected.

**Table 2:** Hormonal Investigations Abbreviations: ACTH, adrenocorticotropic hormone

Parameter	Patient Value	Reference Range
Serum cortisol (8:30 am, nmol/L)	7	133–537
ACTH (ng/L)	270	<50
Serum aldosterone(pmol/L)	48	150-550(supine)
Plasma renin activity	Lab error	
Adrenal cortex antibody	Positive	

Initial management involved administering intravenous fluids, which produced minimal improvement in her blood pressure. Given the suspicion of adrenal insufficiency, intravenous hydrocortisone was started alongside IV fluids. After five days, her calcium levels normalised to 2.6 mmol/L, and her renal function returned to baseline with an eGFR of 76 ml/min/1.73m2.

At her six-month review, she reported significant improvement in quality of life, renal function remained at baseline, haemoglobin levels had normalised, and calcium remained within the normal range (Table 1).

## Discussion

Hypercalcaemia is either PTH-dependent, with normal or elevated PTH, or PTH-independent, with low PTH. Malignancy is the leading cause of PTH-independent hypercalcaemia; other causes include vitamin D toxicity, granulomatous diseases, milk-alkali syndrome, certain drugs (e.g., thiazides), immobilisation, and, rarely, endocrine disorders such as hyperthyroidism and adrenal insufficiency [2,8].

In this case, malignancy was excluded by a normal CT of the chest, abdomen, and pelvis. The myeloma screen was negative. Serum ACE level was within normal limits, and CT imaging showed no lymphadenopathy, ruling out granulomatous diseases. Hormonal assessment confirmed PAI, and calcium levels returned to normal following hydrocortisone replacement and intravenous fluids. The pathophysiology of hypercalcaemia in adrenal insufficiency is thought to involve increased translocation of calcium into the extracellular compartment, coupled with impaired renal excretion of calcium [9]. Volume depletion associated with adrenal insufficiency reduces glomerular filtration rate, thereby decreasing the filtered load of calcium and enhancing tubular reabsorption. Additionally, hypoadrenalism promotes bone resorption, further elevating serum calcium [9]. 

Acute kidney injury may be an initial sign of adrenal insufficiency. Classical biochemical markers, such as hyponatraemia and hyperkalaemia, are not always present and may be misattributed to renal dysfunction, potentially delaying diagnosis [10]. In cases of undiagnosed adrenal insufficiency, acute kidney injury typically does not resolve with fluid therapy alone (without steroid replacement); lack of improvement should prompt consideration of adrenal insufficiency as the underlying cause [10]. In this case, acute kidney injury resolved quickly within five days of IV fluids and steroid replacement. Acute kidney injury in adrenal insufficiency is primarily attributed to reduced renal perfusion and glomerular filtration rate, resulting from extracellular fluid depletion due to mineralocorticoid deficiency and decreased cardiac output from glucocorticoid deficiency [10]. Renal microangiopathy and failure have been reported in some cases of adrenal insufficiency [11]. In this case, vomiting, a symptom of Addison’s disease, likely intensified the patient’s volume depletion, compounding the extracellular fluid loss caused by mineralocorticoid deficiency. This was reflected in her markedly low aldosterone level of 48 pmol/L, which led to diminished renal perfusion and the development of acute kidney injury. Urinalysis was unremarkable, and a renal biopsy was deemed unnecessary, as her renal function promptly improved following treatment with intravenous fluids, hydrocortisone, and fludrocortisone replacement.

There is an isolated case report of adrenal insufficiency presenting with hypercalcaemia and acute kidney injury [12]. However, to our knowledge, adrenal insufficiency with hypercalcaemia, acute kidney injury, and anxiety has not been reported previously. A review of 25 case reports identified a range of neuropsychiatric manifestations associated with Addison’s disease, including delusions (45%), depression (40%), hallucinations (40%), anxiety disorders (24%), irritability and aggression (24%), disorientation (20%), memory impairment (20%), mania (12%), EEG abnormalities (83%), catatonia (8%), and self-mutilation (4%) [13]. In 80% of cases, neuropsychiatric symptoms resolved within one week of initiating cortisone therapy [13]. The pathogenesis of these symptoms remains unclear but may involve electrophysiological, electrolyte, and metabolic disturbances, glucocorticoid deficiency, and increased endorphin levels [13]. In this case, the patient’s anxiety symptoms improved with hydrocortisone replacement, eliminating the need for ongoing citalopram or other anxiolytics.

## Conclusions

This case underscores the importance of considering PAI in the differential diagnosis of PTH-independent hypercalcaemia, particularly when other common causes are excluded. Early recognition and appropriate endocrine evaluation are essential, as prompt initiation of corticosteroid therapy can be lifesaving. Furthermore, this case demonstrates that hypercalcaemia and renal impairment associated with Addison’s disease can resolve promptly with appropriate steroid therapy, and that psychiatric manifestations may also significantly improve, reducing the need for ongoing anxiolytic treatment. Clinicians should maintain a high index of suspicion for Addison’s disease in patients with unexplained electrolyte disturbances and renal impairment to avoid delays in diagnosis and optimise patient outcomes.
